# Primary Hodgkin Lymphoma of the Breast: A Case Report and Literature Review Examining the Use of Different Chemotherapy Regimens

**DOI:** 10.7759/cureus.65541

**Published:** 2024-07-27

**Authors:** Divine Besong Arrey Agbor, Wahib Zafar, Emmanuel A Agyemang, Tochukwu Nzeako, Derek Ugwendum, Sabastain F Forsah, Gauvain Kankeu Tonpouwo, Irene O Pokuaa, Angela Grigos, Jay Nfonoyim

**Affiliations:** 1 Internal Medicine, Richmond University Medical Center, Staten Island, USA; 2 Hematology/Oncology, Richmond University Medical Center, Staten Island, USA; 3 Internal Medicine, Newark Beth Israel Medical Center, Newark, USA; 4 Internal Medicine, Christiana Care Hospital, Newark, USA; 5 Internal Medicine, Capital Health Medical Center, Trenton, USA; 6 Pulmonary and Critical Care, Richmond University Medical Center, Staten Island, USA

**Keywords:** abvd chemotherapy, primary hodgkin breast tumor, nodular sclerosis breast hodgkin lymphoma, classical breast hodgkin lymphoma, breast hodgkin lymphoma

## Abstract

Primary classic Hodgkin lymphoma (HL) of the breast is a rare type of breast disease. The diagnosis is mostly confirmed by an excisional biopsy. The first line of treatment commonly used for Hodgkin lymphoma is doxorubicin, bleomycin, vinblastine, and dacarbazine (ABVD). Our case report is about a 48-year-old lady who was diagnosed with bilateral breast Hodgkin lymphoma following an excisional biopsy and was treated with brentuximab vedotin plus doxorubicin, vinblastine, and dacarbazine (BV-AVD). The patient responded positively after the initiation of the regimen. There is scarce data on the classic Hodgkin lymphoma of the breast, and even with the wide use of first-line treatment using ABVD, the disease is still difficult to manage. Hence, patients with breast masses should be screened for classic HL of the breast, and larger studies are needed to establish specific treatment guidelines concerning HL of the breast to prevent relapse and other complications.

## Introduction

Hodgkin lymphoma (HL) has two types, namely the classic Hodgkin lymphoma (cHL) and nodular lymphocyte predominant Hodgkin lymphoma (NLPHL). The cHL is more common and made of the following subtypes: nodular sclerosing, mixed cellularity, lymphocyte-depleted, and lymphocyte-rich. Primary breast Hodgkin lymphoma, or metastatic breast disease, is a rare condition. The incidence of the primary form ranges from 0.04% to 0.5% of all breast neoplasms, while the metastatic form has an incidence of about 0.07% [1,2]. The breast is an unfamiliar site for extranodal Hodgkin lymphoma. Therefore, very few cases have been reported in the literature.

The diagnosis of lymphomas is traditionally established by histology on a surgical biopsy sample with an appropriate panel of immunostains. However, in recent years, fine needle aspiration (FNA) has reached an important and definite role in the diagnosis of lymphomas and reactive lesions in both lymph nodes and extranodal sites with the use of ancillary techniques such as flow cytometry and immunocytochemistry. FNA assisted by flow cytometry can provide specific diagnoses of lymphoid breast lesions [3]. The combination of these techniques differentiates reactive from neoplastic processes, with a sensitivity of 90% and specificity of 100%. Thus, it simplifies patient management, preventing unnecessary biopsies [4]. Diagnostic criteria for primary lymphoma of the breast are several: the presence of adequate tissue for pathological evaluation, close association between breast tissue and lymphomatous infiltrate, and no evidence of concurrent widespread disease or preceding extramammary lymphoma. The presence of ipsilateral axillary nodal involvement does not constitute grounds for rejection only if both lesions developed simultaneously [5].

The treatment of Hodgkin lymphoma of the breast consists of radiation therapy, targeted therapy, immunotherapy, surgery, and chemotherapy with stem cell transplant. The classification of treatment is based on the disease's progression, including early favorable classic HL, early unfavorable classic HL, advanced HL, and recurrent classic HL [6]. Concerning chemotherapy, there are different combination regimens approved depending on the patient's clinical state.

Our case was that of a middle-aged woman who was diagnosed with bilateral breast Hodgkin lymphoma following an excisional biopsy and was treated with brentuximab vedotin plus doxorubicin, vinblastine, and dacarbazine (BV-AVD). We shall also compare cases of classic breast Hodgkin lymphoma that have been reported. Considering the scarcity of data about breast Hodgkin lymphoma, there is need for future investigation to establish Hodgkin lymphoma as a differential in patients presenting with a breast mass and subsequent appropriate management.

## Case presentation

We present the case of a 48-year-old female who presented with shortness of breath, body weakness, chest pain, loss of appetite, back pain, and bilateral breast pain for a period of one week. Her past medical history is not significant. She does not smoke cigarettes, has no illicit drug intake, and has no alcohol consumption. Her clinical examination on admission revealed an altered general state, as well as prostration and hypertension. There were palpable lymph nodes in the bilateral axillary and cervical regions. She had a palpable, firm, 5-6 cm mobile mass adjacent to the nipple of the right breast with skin dimpling. Her laboratory results showed leukocytosis, anemia, and thrombocytosis. There was a mildly elevated reticulocyte count and lactate dehydrogenase level. The tumor markers 'CEA, CA15-17, CA27-29, BhCG, AFP' were within normal ranges, as shown in Table 1 below.

**Table 1:** Showing the different laboratory results for our patient.

Test	Result	Normal Range/Units
White blood count	16.6	4-11.2 K/UL
Red blood cell count	3.57	3.90-5.20 m/ul
Hemoglobin	7.1	11.2-15.7 g/dl
Platelet count	628	150-400 k/ul
Reticulocyte count	2.92	0.9-2.5%
Lactate dehydrogenase	384	120-240 u/l
Haptoglobin	273	43-212 mg/dl
Alkaline phosphatase	155	46-116 U/L
Alpha fetoprotein	2.6	0.5-77 ng/ml
Carcinoembryonic antigen	<0.5	0-2.5 ng/ml
CA15-3 antigen	14	<32 u/ml

**Figure 1:**
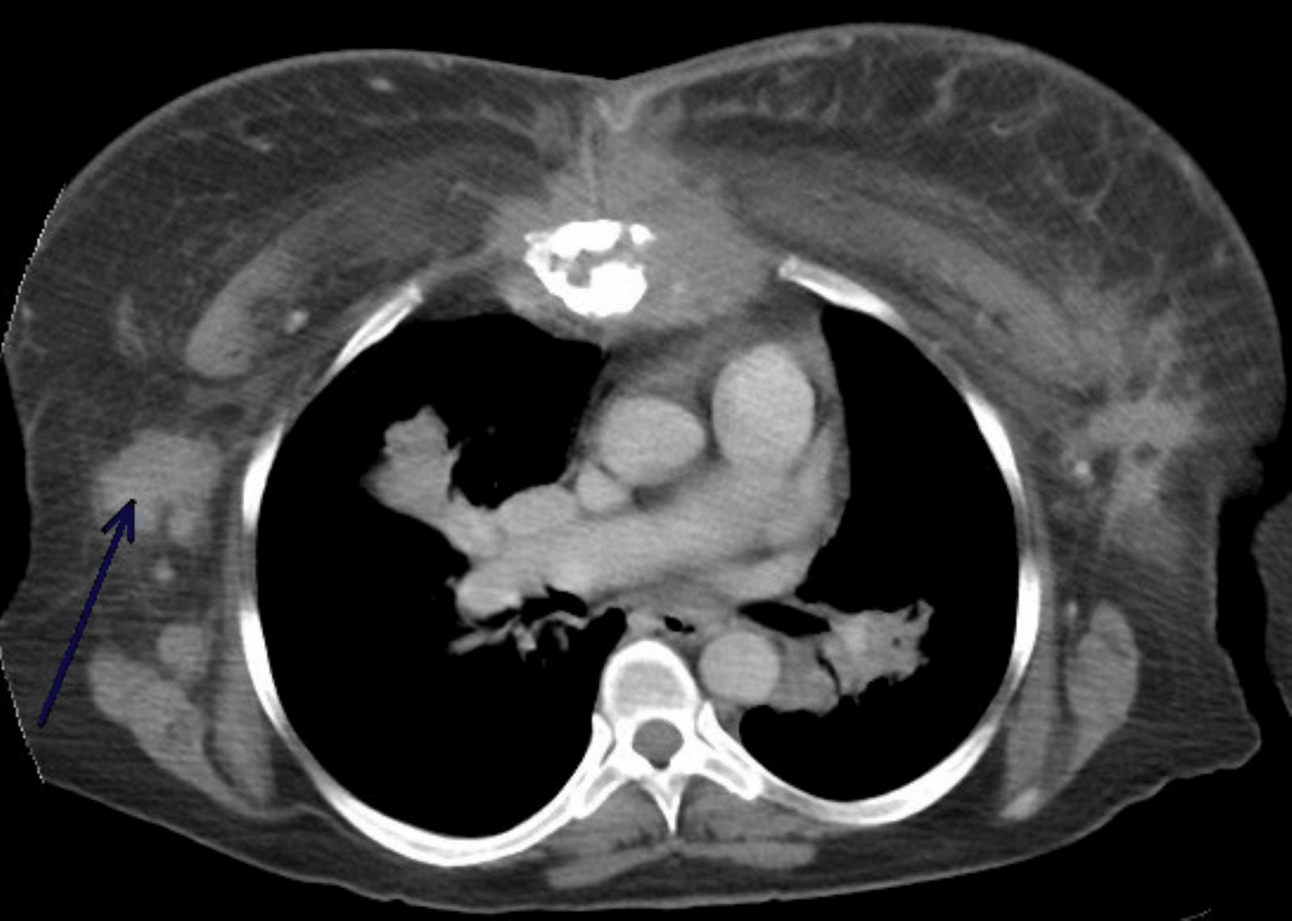
Computer tomography (CT) of the chest shows bilateral breast masses consistent with possible breast cancer.

Imaging done included computed tomography '(CT) of the chest/abdomen/pelvis' which showed a probably primary cancer of the right upper breast with significant metastasis and breast findings of clear malignancy with lesions on the lungs, chest wall, and axillary regions. In addition, a mass was seen on the left breast, malignant adenopathy below the diaphragm with splenomegaly and direct invasion into the sternum and right upper ribs, as well as bone metastasis at the L4 vertebral body, as illustrated in Figure 1.

**Figure 2:**
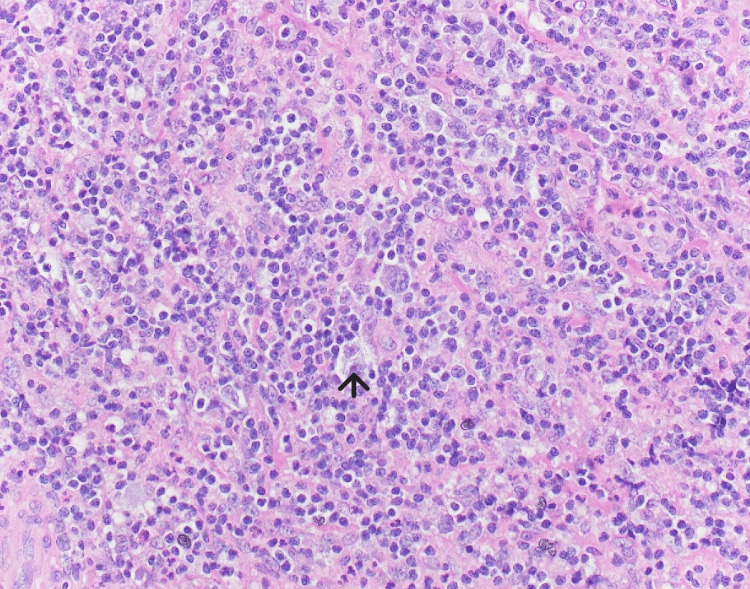
Breast pathology results demonstrate Reed Sternberg mononuclear cells confirming Hodgkin lymphoma.

Pathology results confirmed classic nodular sclerosing Hodgkin lymphoma. Hematoxylin and eosin showed fragments of tissues with fibrosclerosing changes and a marked inflammatory background, inflammatory infiltrates composed of small lymphocytes, neutrophils, and some eosinophils with scattered and few sheets of large abnormal cells. The large abnormal cells had oval to occasionally multilobate nuclei, vesicular chromatin nuclei, and abundant cytoplasm corresponding to Reed Sternberg mononuclear variants, as demonstrated in Figure 2.

Immunostaining revealed large abnormal cells positive for PAX5, CD30, MUM1, PDl1, CD15 (subset), CD20. The BOB1 were positive for a subset of small B cells. The patient was started on brentuximab vedotin plus doxorubicin, vinblastin, and dacarbazine (BV-AVD) for six cycles. In addition, allopurinol 300 mg and ondansetron 8 mg were ordered to prevent tumor lysis syndrome and vomiting, respectively. Evolution was marked by significant reductions in symptoms. Positron emission tomography (PET)-CT of the skull to mid-thigh post-chemo showed interval development of the left breast lesions and axillary lymph nodes.

## Discussion

Search strategy

We did a comprehensive literature search using electronic databases, such as PubMed, PubMed Central, MEDLINE, and Google Scholar. Keywords such as “breast Hodgkin lymphoma,” “classic Hodgkin lymphoma,” and “nodular sclerosis breast lymphoma” were used to look for our studies of interest.

Eligibility criteria (inclusion and exclusion)

Our search included studies that provided details into classic breast Hodgkin lymphoma. We particularly selected articles published in peer-reviewed journals, and those were mostly case reports. We excluded non-English articles and those that were not available in full text.

Data extraction

Relevant information was taken from selected articles, including diagnostic criteria, different treatments, and outcomes. We focused on synthesizing the different presentations, diagnoses, and treatments.

Quality assessment

The quality of selected studies was assessed based on the reliability of the data sources, methodological protocol, and how useful it was to our topic. Only case reports were found in our search.

Data synthesis 

The extracted information was organized to provide a cohesive narrative that evaluates existing breast Hodgkin lymphoma and a new case. Comparative analyses noted the different approaches’ similarities, differences, strengths, and limitations.

Review framework

**Figure 3:**
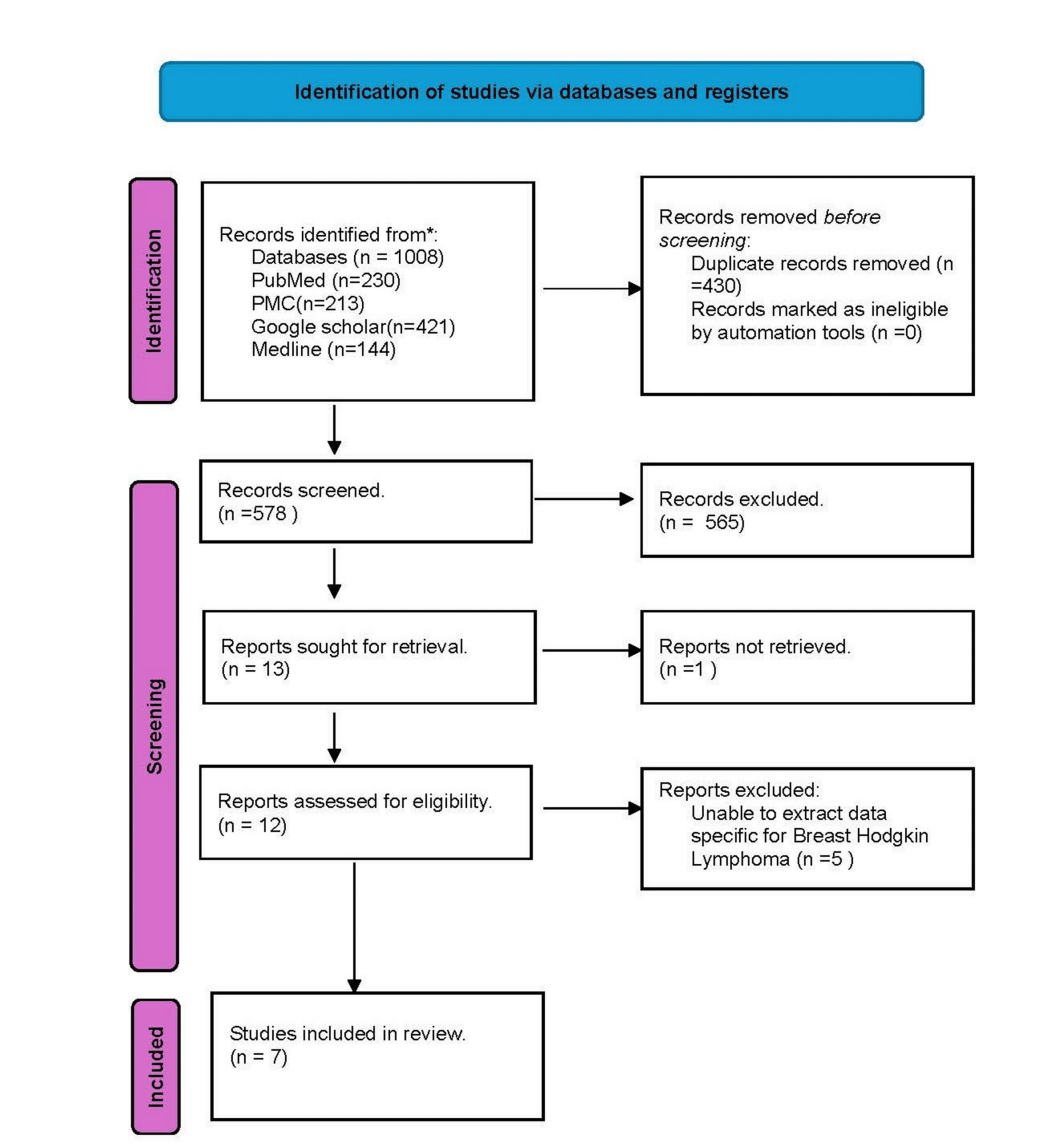
Prisma flow chart for the studies retained.

Our literature search adheres to the Preferred Reporting Items for Systematic Reviews and Meta-Analyses (PRISMA) guidelines [7] as demonstrated in Figure 3, ensuring transparency in selecting and processing important findings.

Our search found a total of one thousand and eight articles. We recorded four hundred and thirty duplicates, which were discarded using EndNote. We screened five hundred and seventy-eight articles manually by titles and content, ending up with thirteen articles because five hundred and sixty-five articles were eliminated. In all, we retained seven articles that reported 24 cases. Seventeen of the cases were mentioned in one of the papers from 1928 to 2016. We had data for the additional seven cases, which are illustrated in Table 2. It was found that most of the patients were diagnosed with classic nodular sclerosis Hodgkin lymphoma and were on an ABVD regimen, as demonstrated in Table 2.

**Table 2:** Showing different studies about breast Hodgkin lymphoma. HL: Hodgkin lymphoma; ABVD: doxorubicin, bleomycin, vinblastine, and dacarbazine; EBV: Epstein-Barr virus

Author	Diagnosis	Treatment
Mario Faenza et al. [8]	Primary nodular sclerosing variant HL of the breast	ABVD
Ingrid Marton [9]	EBV-positive primary HL of the breast	Surgery, No chemotherapy
Yashpal Modi et al. [10]	Nodular sclerosis HL of the breast	ABVD
Charles Osuji et al. [11]	Nodular sclerosing HL of the breast	ABVD
Zarnescu et al. [12]	Nodular sclerosis HL of the breast	Lumpectomy and lymphadenectomy, No chemotherapy
Christopher Hoimes et al. [13]	Nodular sclerosis HL of the breast	ABVD
Eleni Thodou et al. [14]	Nodular sclerosis HL of the breast	ABVD

Classic Hodgkin lymphoma is a monoclonal tumor composed of Reed-Sternberg cells combined with a different population of nonneoplastic immune cells usually arising in the lymph nodes [15]. Primary Hodgkin lymphoma of the breast is a very rare occurrence. The lymphatic association of the breast parenchyma can be an expression of either a primary illness or a secondary illness, in which the infiltration of the breast is due to a systemic disease or a disease recurrence. According to a recent review, 24 cases of primary Hodgkin lymphoma of the breast have been reported since first described by Kueckens in 1928 [16,17].

Refractory cases of classic breast HL

Eleni Thodou et al. [14] described a case of a 57-year-old lady who was evaluated for symptoms of lymphedema, inflammation, and firmness, along with an orange peel-like skin appearance in her right breast. A palpable lump in the right axilla preceded the breast lesion. The axillary lymph node and the breast lesion were the targets of US-guided FNA. Large malignant lymphoid cells were seen scattered throughout a reactive population of eosinophils and tiny lymphocytes. Large nuclei with a noticeable nucleolus and copious pale basophilic cytoplasm were present in the cancerous cells. A few of them were multinucleated, and a significant portion of them were binucleated with Reed-Sternberg cell characteristics. Immunostains were performed on some of the Hemacolor-stained slides. The cancerous cells were positive for CD30 and CD15. The patient had an ABVD regimen (doxorubicin, bleomycin, vinblastine, and dacarbazine). The axillary tumor was still perceptible one month prior to the completion of all ABVD cycles. After being restaged, a PET-CT scan revealed elevated fluorodeoxyglucose (FDG) uptake in the right axillary and breast regions and a notable rise in the lymph node block. The patient's condition was diagnosed as primary refractory HL, and chemotherapy with cisplatin, cytarabine, and dexamethasone (DHAP regimen) was started. In order to move on with autologous transplant consolidation after one cycle of DHAP with mild toxicity (grade II without febrile neutropenia), a fresh CT scan was carried out to assess for chemosensitivity. A cycle of dexamethasone, ifosfamide, cisplatin, and etoposide (DICE) plus brentuximab vedotin (BV), a recently approved anti-CD30 antibody-drug conjugate, was selected after the CT scan showed no improvement. The patient was hospitalized because of febrile neutropenia, anemia, and pyelonephritis, and grade IV hematological damage was noted. She was admitted to the hospital for a week, during which she had red blood cell and platelet transfusions, granulocyte colony-stimulating factor (GCSF) injections, intravenous fluid, and antibiotics, and gradually recovered before being discharged from the facility. She was further managed with BV monotherapy at a dose of 1.8 mg/kg every three weeks. There was complete remission of lymphadenopathy after four infusions, the disappearance of the breast lesion, and a significant improvement of the lymphedema.

A case was reported by Yashpal Modi et al. [10] about a 25-year-old woman who initially came for assessment of a palpable left breast lump that had been present for a month and was later diagnosed as nodular sclerosing HL from the left axillary lymph node biopsy. She was classified as stage 2B at the time of her first diagnosis. She only had two rounds of the chemotherapy regimen, which included vinblastine, dacarbazine, bleomycin, and Adriamycin, before being lost to follow-up because she refused to comply with the prescribed course of treatment. Upon inspection, she had a hard, fixed mass with a diameter of 2 cm near her left nipple. Studies using ultrasounds showed no signs of calcifications but rather an asymmetric hypoechoic density over the left nipple and mild deformation of the surrounding breast tissue. Cytology using fine-needle aspiration raised a suspicion for cancer. After that, a core biopsy was performed using ultrasound guidance. Histopathology showed nodular sclerosing HL with CD30 and CD15-positive neoplastic Reed-Sternberg cells. After undergoing a fluorine-18 fluorodeoxyglucose positron emission tomography/computed tomography (FDG-PET/CT) scan for restaging, the left cervical, left axillary, and left inguinal regions were found to have FDG-avid hypermetabolic lymphadenopathy. Because of the disease's advancement and her previous non-compliance, she returned to her original regimen.

The recurrence of classic HL was noticed in patients taking the standard doxorubicin, bleomycin, vinblastine, and dacarbazine (ABVD), as reported by Eleni Thodou et al. [14] and Yashpal Modi et al. [10], which was different from our case, who was on brentuximab, vedotin plus doxorubicin, vinblastin, and dacarbazine (BV-AVD). This difference could be explained by the fact that our patient was diagnosed with classical Hodgkin lymphoma of the bilateral breasts. On the other hand, clinical improvement has been observed in some patients who were diagnosed with classical HL and were on the ABVD regimen, as reported by Mario Faenza et al. [8] and Charles Osuji et al. [11]. This difference in findings can be due to varying stages of the disease at diagnosis or treatment.

The different methods of diagnosis used in cHL

The diagnosis of classic Hodgkin lymphoma commences with history-taking and clinical examination. The symptoms constitute fever, heavy drenching sweat, weight loss, fatigue, palpable lymph nodes, and breast masses. The best way to diagnose cHL is an excisional biopsy, and the use of immunochemistry showed proteins CD15 and CD30. Fine needle biopsy should be used with adjunct methods because it does not collect the entire sample to be analyzed. Other additional tests performed include complete blood count, complete metabolic panel, erythrocyte sedimentation rate, lactate dehydrogenase, liver function test, hepatitis B, and hepatitis C. The imaging used in staging the disease is positron emission tomography (PET) and computed tomography (CT) [18-20]. From our review, all the patients presented with palpable breast masses and multiple lymph nodes. A fine needle aspiration biopsy done in the patients reported by Mario Faenza et al. [8] and Zarnescu et al. [12] was inconclusive, and the diagnosis was later confirmed by an excisional biopsy. This supports the fact that FNA should always be accompanied by an ancillary test like flow cytometry [8,12]. However, Christopher Hoimes et al. [13] carried out US-guided FNA on breast mass, and the patient had definitive results of nodular sclerosis classical Hodgkin lymphoma. This difference may be due to the fact that the patient had a single and small lesion. Eleni Thodou et al.'s [14] results showed that cHL was diagnosed using FNA with flow cytometry, which is consistent with the standard recommendations [14,19]. Furthermore, Yashpal Modi et al. [10], Charles Osuji et al. [11], and Ingrid Marton et al. [9] carried out an excisional biopsy for their patients, and the results were conclusive, again similar to the standard of practice. Interestingly, Ingrid Marton et al. [9] was the only study that reported Ebstein-Barr virus-associated classical Hodgkin lymphoma. PET-CT scan was well utilized for staging by all the studies retained.

Treatment regimen used

The standard Hodgkin lymphoma treatment comprises systemic therapies, radiation therapy, and high-dose chemotherapy with stem cell rescue. The systemic therapies are chemotherapy, targeted therapy, and immunotherapy. The use of high-dose chemotherapy with stem cell rescue could be autologous bone marrow transplant, autologous stem cell transplant, hematopoietic cell transplant, and high-dose therapy with autologous stem cell rescue [21,22]. The most commonly used chemotherapy regimens are ABVD: doxorubicin, bleomycin, vinblastine, and dacarbazine; AVD: doxorubicin, vinblastine, and dacarbazine; BV+ AVD: brentuximab vedotin + AVD; and escalated BEACOPP: bleomycin, etoposide, doxorubicin, cyclophosphamide, vincristine, procarbazine, and prednisone [23]. The treatment of classic Hodgkin lymphoma is further divided according to stages as illustrated in Table 3.

**Table 3:** Different treatment regimens used in cHL. cHL: classic Hodgkin lymphoma; ABVD: doxorubicin, bleomycin, vinblastine, and dacarbazine; AVD: doxorubicin, vinblastine, and dacarbazine

cHL stage /category	Treatment
Early cHL (stage I-II)	Combination therapy (both chemotherapy and radiation or chemotherapy alone)
Advanced cHL(III-IV)	ABVD or brentuximab vedotin + AVD
Refractory cHL	Brentuximab vedotin, or brentuximab vedotin + bendamustine or brentuximab + nivolumab or DHAP (dexamethasone, cisplatin, high dose cytarabine) or pembrolizumab or GVD (gemcitabine, vinorelbine, liposomal doxorubicin), and others
Older adults with cHL	2 cycles ABVD or AVD, or 4 cycles CHOP (cyclophosphamide, doxorubicin hydrochloride, vincristine sulfate, and prednisone) + radiation
Older adults stage I-IV	2 cycles of ABVD or AVD or chemotherapy with brentuximab vedotin followed by AVD or chemotherapy with brentuximab vedotin + dacarbazine or six cycles of CHOP with or without radiation
Older adults with refractory cHL	Chemotherapy with bendamustine, chemotherapy with brentuximab, radiation therapy, or immunotherapy with nivolumab or pembrolizumab

From our search, we found that Mario Feanza et al. [8], Charles Osuji et al. [11], and Christopher Hoimes et al. [13] reported that their cases of breast Hodgkin lymphoma were treated with an ABVD regimen, which resulted in complete remission. These findings support the management of early classic Hodgkin lymphoma. Secondly, Yashpal Modi et al. [10] and Eleni Thodou et al. [14] mentioned their cases with classic Hodgkin lymphoma of the breast developed refractory disease from ABVD. These differences concerning Yashpal Modi et al. [10] could be explained by the fact that the patient was non-compliant with her treatment given that she was young. Regarding Eleni Thodou et al. [14] findings, it was noted that the patient was started on ABVD. Due to the progression of the disease, the regimen was changed to dexamethasone, cisplatin, and cytarabine (DHAP) and subsequently to dexamethasone, ifosfamide, cisplatin, and etoposide (DICE) in combination with brentuximab vedotin (BV). There was the persistence of the disease whereby the treatment was finally replaced with BV monotherapy at a dose of 1.8 mg/kg every three weeks and total remission was achieved after some time [14]. This plan of action is consistent with the standard of care for refractory classical Hodgkin lymphoma [22,23]. Our case was diagnosed with bilateral breast HL, and treatment was initiated with brentuximab, vedotin plus doxorubicin, vinblastin, and dacarbazine (BV-AVD) for six cycles. A PET-CT scan showed the progression of the disease, and she continues to follow up with her oncologist. This management is similar to one of the more frequently utilized regimens.

Lastly, Ingrid Marton et al. [9] and Zarnescu et al. [12], reported cases with classical Hodgkin lymphoma of the breast were managed with surgical intervention and were followed up with an outpatient oncologist without mentioning the kind of chemotherapy given or clinical evolution.

## Conclusions

Primary classical Hodgkin lymphoma of the breast is an unusual breast disease, and very few cases have been reported. Our case was a rare presentation of bilateral breast classical Hodgkin lymphoma with refractory symptoms and was treated with a regimen of BV-AVD. In addition, we found that excisional biopsy is the standard diagnosis for cHL of the breast with adjunct immunochemistry that is positive for CD15 and CD30 proteins. The first line treatment mostly used among patients was doxorubicin, bleomycin, vinblastine, and dacarbazine (ABVD), and brentuximab vedotin was used as a single chemotherapy for refractory cases of cHL with total remission. There exists scarce information about breast classical Hodgkin lymphoma, and though the first-line treatment of ABVD is widely used, the disease is still difficult to manage. Thus, there is a need for larger studies to be carried out to determine more specific treatments for breast Hodgkin lymphoma and to prevent relapse with other complications.
